# A Retrospective Review of Silver Sulfadiazine Use in Patients With Sulfa-Allergic Burns at a Regional Burn Center

**DOI:** 10.7759/cureus.84360

**Published:** 2025-05-18

**Authors:** William West, Kristina T Buller, Mariel McArthur, Benjamin Wright, Michael Bodnar, Jenna C Marek, David J Smith, Jake Laun

**Affiliations:** 1 Morsani College of Medicine, University of South Florida, Tampa, USA; 2 Department of Plastic Surgery, University of South Florida, Tampa, USA

**Keywords:** burn and wound ointment, burn wound care, history of allergy, silver sulfadiazine, sulfa drug

## Abstract

Background

Silver sulfadiazine is the most common topical antimicrobial used in burn management. The intent of this retrospective review is to examine the safety of silver sulfadiazine use in documented sulfa-allergic burn patients at a level one trauma hospital and regional burn center.

Methods

Institutional review board approval was obtained for electronic chart review of all patients who presented to a level one burn center between 2018 and 2022. Charts were reviewed, and exclusion criteria were applied. Patients included in the study had documented burns treated by burn team providers, application of topical silver sulfadiazine for primary burn management, and a documented sulfa allergy. Chart review identified 71 patients who met the stated criteria out of 2,654 patients treated over a five-year period.

Results

None of the 71 patients suffered an adverse reaction to silver sulfadiazine used for primary burn care. There were no documented systemic or anaphylactic reactions, hives, or additional adverse reactions after administration. No cessation of the medication was documented due to intolerance.

Conclusions

It is reasonable for providers to have a discussion with their sulfa-allergic burn patients about the risks, benefits, and alternatives of using silver sulfadiazine if they feel its use is indicated.

## Introduction

Burn injuries are commonly encountered wounds, where the source of injury can be friction, cold, heat, radiation, chemical, or electric in nature [1]. Flame burns are still the most common burn etiology, followed by scald. Regardless of the burn etiology, the resultant effect is a dysregulated inflammatory host response, which requires specialized treatment in regard to surgical care and wound management [2].

Although multiple dressings are utilized in wound care, silver sulfadiazine is the most common topical antimicrobial used in burn management [3]. It is applied to second- and third-degree burns to prevent wound infection and assist with wound healing. Burn wound dressings typically consist of 1% silver sulfadiazine as standard care. The mechanism of action of silver sulfadiazine involves the dissociation of silver and sulfadiazine, leading to the binding of silver to bacterial DNA. The importance of sulfadiazine lies in its ability to dissociate from silver slowly and continuously over time, making silver sulfadiazine function as a reservoir for obtainable silver ions in the wound. This makes silver sulfadiazine a more effective antibiotic than other silver-containing compounds in burn wounds [4]. Each gram of 1% silver sulfadiazine cream contains 10 mg of micronized silver sulfadiazine. Consequently, current literature recommends avoiding the use of silver sulfadiazine in burn patients with documented sulfa allergies, and the FDA lists hypersensitivity to silver sulfadiazine or any of its ingredients as a contraindication to its use [5-7]. Any side effect to sulfa drugs is commonly documented as a ‘sulfa allergy’ in the patient’s medical chart, resulting in patient avoidance of any sulfonamide-containing drug due to a presumed ‘sulfa allergy’. However, epidemiological, in vitro, and skin test studies have pointed out that true allergy secondary to cross-reactivity of sulfonamide antibiotics and non-antibiotics is unlikely to occur [8]. It is the intent of this retrospective review to examine the safety of silver sulfadiazine use in documented sulfa-allergic burn patients at a level one trauma hospital and regional burn center.

## Materials and methods

The study was conducted at the University of South Florida, Tampa, FL. We performed a retrospective review of all burn patients treated at a level one trauma and regional burn center over a five-year period, ranging from 2018 to 2022. Inclusion criteria for charts reviewed were documented burns by a burn team physician provider, use of topical silver sulfadiazine for burn treatment, and documented sulfa allergy in the chart. Exclusion criteria were patients without a documented sulfa allergy or those not treated with silver sulfadiazine. At our institution, burn wounds are cleaned with hydrotherapy, including the removal of all debris and old dressing, before silver sulfadiazine (Silvadene®, Pfizer, New York, NY) is applied to affected areas of the body, excluding the face, ears, and genitalia, twice daily and covered with a sterile gauze. Bacitracin and polymyxin B (Polysporin®, Johnson & Johnson, New Brunswick, NJ) are used for affected areas of the face and genitalia, while mafenide acetate (Sulfamylon®, Viatris, Canonsburg, PA) is used for affected areas of the ears. Antihistamines are not routinely given as part of standard burn care.

**Figure 1:**
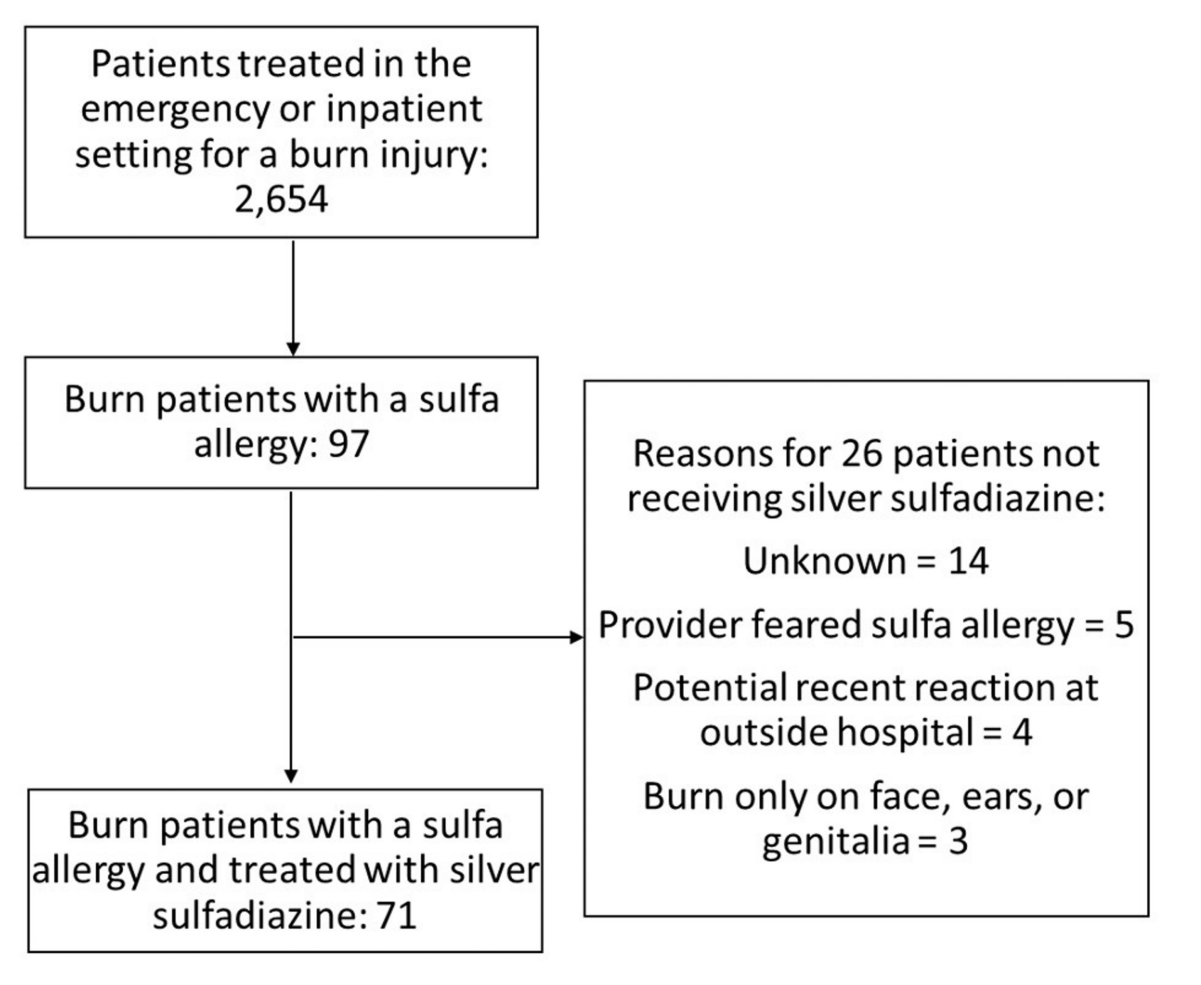
Flowchart showcasing the inclusion and exclusion of patients in the study

Between 2018 and 2022, a total of 2,654 patients were identified who were treated in the emergency or inpatient setting for a burn injury. Of these patients, 97 had a documented sulfa allergy, with 26 not receiving silver sulfadiazine and therefore being excluded from this study. In 14 of these 26 patients, the reason for not using silver sulfadiazine could not be determined based on retrospective chart review. In five cases, the provider decided to use an alternative topical antimicrobial out of fear of a reaction. Four patients were not given silver sulfadiazine at our facility due to what the patients perceived as an allergic reaction from silver sulfadiazine treatment at an outside hospital for the same burn, as they were treated at our hospital. Finally, three patients did not receive silver sulfadiazine because their burn was solely on their face, ears, or genitalia. The remaining 71 patients were administered silver sulfadiazine for primary burn treatment (Figure 1). Patient age and gender, burn etiology and total body surface area (TBSA), sulfa allergy severity, and silver sulfadiazine treatment duration and adverse reactions were all obtained through direct review of patient charts. Descriptive statistics were performed using Microsoft Excel (Microsoft Corp., Redmond, WA), with categorical variables presented as frequencies and continuous variables presented as the mean ± standard error of the mean.

The institutional review board (IRB) deemed our observational, retrospective study exempt (approval number: STUDY003178). Patient data were kept in a secure drive per the IRB protocol. All patients treated at our institution sign an informed consent that their unidentified patient information may be used for research purposes.

## Results

**Figure 2:**
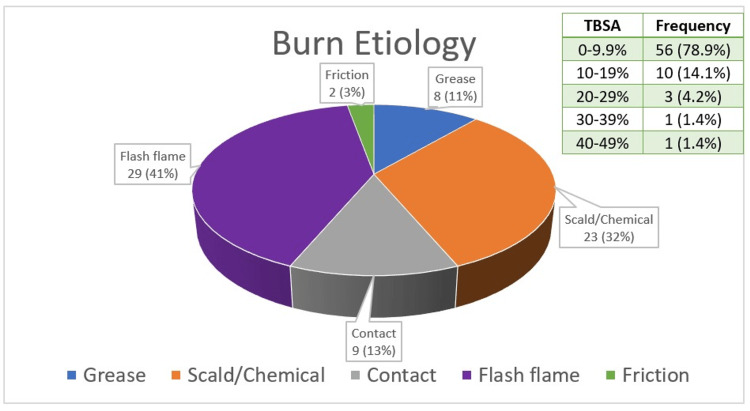
The etiology of burns for patients included in this study was primarily flash flame and scald. Total body surface area (TBSA) of burns noted ranged from 0.5% to 43.0%.

Out of the 71 burn patients with a documented sulfa allergy who were treated with silver sulfadiazine, 35.2% had a low-severity allergy to sulfa drugs reported in their medical history, such as rash, hives, or itching. 8.5% had a severe allergy reported in their history, such as anaphylaxis. 56.3% had an allergy to sulfa drugs documented in their medical history, with no details on allergy symptoms. Silver sulfadiazine application locations spanned the entire body, including the head and neck, anterior torso, posterior torso, and extremities. Days of silver sulfadiazine use ranged from one to 263 days, with a mean duration of 19.5 ± 4.5 days. The age of patients included in the study ranged from 15 to 91 years, with female gender predominance (63.4%) (Table 1). The etiology of burns for patients included in this study was primarily flash flame (40.8%) and scald (32.4%), and the TBSA of burns noted ranged from 0.05% to 43% with an average of 6.5% ± 0.9% (Figure 2).

**Table 1:** Age-wise and gender-wise distribution of this patient population are summarized, with female predominance noted

Age-wise and gender-wise distribution of patients			
Age range (years)	Male	Female	Total
Age 10-19	2	0	2
Age 20-29	5	5	10
Age 30-39	5	6	11
Age 40-49	5	9	14
Age 50-59	4	11	15
Age 60-69	0	8	8
Age 70-79	3	4	7
Age 80-89	1	2	3
Age >90	1	0	1
Total	26	45	71

None of the 71 patients with a documented sulfa allergy who were treated at our institution with topical silver sulfadiazine suffered an adverse reaction to silver sulfadiazine used for primary burn care. In these 71 patients, there were no documented systemic or anaphylactic reactions, hives, or additional adverse reactions after administration. No cessation of the medication was documented due to intolerance at our institution. Four of the patients in the excluded group of 26 patients reported what they thought were allergic reactions to silver sulfadiazine prescribed at an outside hospital shortly before presentation to our facility. One patient reported experiencing skin burning from three days of silver sulfadiazine use before presenting at our hospital. Another patient reported malaise, myalgias, nausea, and vomiting after receiving silver sulfadiazine at an outside hospital. A third patient reported intense pain and believed it was due to silver sulfadiazine. Finally, a patient received silver sulfadiazine and hydrocodone at an outside hospital and two hours later developed shortness of breath, wheezing, and hives. They were treated for an anaphylactic reaction with diphenhydramine, steroids, and epinephrine. The next day, they developed shortness of breath and wheezing and were treated for an asthma exacerbation. They subsequently presented to our hospital.

## Discussion

Silver sulfadiazine is commonly used for burn care due to its antimicrobial properties, which prevent burn infections and promote healing [9]. In addition, silver sulfadiazine use in burn care has been demonstrated in some studies to decrease a patient’s length of stay and be associated with fewer hospital-acquired complications [10]. Although there are known documented adverse reactions with silver sulfadiazine use, such as leukopenia, this is typically a self-limited phenomenon that does not increase the incidence of infectious complications with discontinuation of use [11]. Silver sulfadiazine use in patients with documented sulfa allergy is cautioned. The FDA does list previous reaction to silver sulfadiazine as a contraindication to its use [5]. However, drug monographs notoriously vary from source to source and have been shown to vary widely for sulfadiazine, ranging from contraindication in patients with known sulfonamide hypersensitivity to caution given rare reports of adverse reactions [6]. One source gave sulfadiazine a category rating of mostly unsafe for use in patients with sulfonamide hypersensitivity, with a warning for cross-reactivity, but this was based on data from two case reports [12, 13]. A handful of previous studies have found potential allergic reactions to topical silver sulfadiazine use in burn patients, including two case reports of erythema and edema that resolved with discontinuation [13, 14], a case report of nephrotic syndrome that resolved with immunosuppressive medication [15], and a case report of topical silver sulfadiazine potentially triggering a subsequent outbreak of generalized rash upon initiation of trimethoprim-sulfamethoxazole that resolved with steroid treatment [12]. One study found that in a group of 45 patients treated with topical silver sulfadiazine, 90% developed antibodies to sulfadiazine, although no allergic reactions developed clinically [16]. However, its safety has been alluded to in sulfa-allergic patients due to its limited systemic penetrance with topical application, and no case report has ever documented anaphylaxis [17].

In this retrospective review, we have identified 71 patients with documented sulfa allergies who suffered no apparent complications with topical silver sulfadiazine application for burn care use. There were four patients in the excluded group of 26 patients with a sulfa allergy not treated with silver sulfadiazine at our hospital who reported an allergic reaction due to silver sulfadiazine application at an outside hospital. Due to the circumstances, it is unclear whether their experiences constituted actual allergic reactions to silver sulfadiazine. In two of these four cases, the patient’s subjective experience of an “allergic reaction” was pain at the application site. It is likely that these two cases were related to the burn itself and not silver sulfadiazine. The third case involved malaise, myalgias, nausea, and vomiting, which would not be a sign of an allergic reaction to silver sulfadiazine. In the fourth patient, who developed shortness of breath, wheezing, and hives, it may have been a reaction to concurrent hydrocodone administration or part of the asthma exacerbation for which the patient was subsequently treated. In these four cases, the patients elected to undergo another form of wound care at our institution after consultation with the provider. Providers should abide by the ethical principle of nonmaleficence, but patient-reported allergies have been shown to often be incorrect [18, 19]. A discussion between provider and patient involving the informed consent process and allowing for patient autonomy is likely the best course of action when deciding on topical silver sulfadiazine use in patients with reported allergies to previous exposures.

Providers and patients are understandably most concerned about prescribing silver sulfadiazine in burn patients when documented sulfa allergy is severe, such as anaphylaxis. Six of the 71 included patients (8.5%) had a severe sulfa allergy, and none had any reaction to silver sulfadiazine. Five of the 26 excluded patients (19.2%) had a severe sulfa allergy. For three of these patients, we were not able to determine why they never received silver sulfadiazine at our institution. A fourth patient with a documented severe allergy did not receive silver sulfadiazine because a provider at our institution was concerned about the documented history of anaphylaxis with sulfonamides, which is the exact situation we hope this study will address. The fifth patient with a documented severe allergy was not prescribed silver sulfadiazine because of the shortness of breath, hives, and wheezing after silver sulfadiazine and hydrocodone administration at an outside hospital. Again, it is unclear if this was due to silver sulfadiazine or hydrocodone.

Limitations

This study had several limitations. First, the study relied on self-reported allergies. Patients may have incorrectly stated they had allergies to sulfa drugs, causing an overestimation in the number of patients with true sulfa allergies. Previous studies have demonstrated that 28-40% of reported allergies can be removed from patient records after pharmacist interviews because they are actually intolerances or adverse effects and not true allergies [18, 19]. Our study likely had a high number of these patients, as 40/71 (56.3%) included patients and 10/26 (38.5%) excluded patients reported that they did not know their actual reaction to sulfa drugs. Another limitation is the presence of the 26 excluded patients who had a sulfa allergy but did not receive silver sulfadiazine at our institution. This group of patients could theoretically be the most likely to have an allergic reaction to the silver sulfadiazine, especially as the percentage of documented anaphylaxis in these patients was over 10% greater than in the included group. A third potential limitation is the limited length of stay by many of the patients. Over half the included patients had a burn TBSA of less than 10%. These patients are often only hospitalized for one or two days, potentially not providing enough time to develop a reaction to silver sulfadiazine. However, there were no documented reactions in the follow-up notes when patients were evaluated in the burn clinic. The final limitations are due to the retrospective nature of the study. We were unable to record the TBSA of silver sulfadiazine application. However, this number can be estimated as being no lower than 8% less than the reported TBSA, as the only burned areas not treated with silver sulfadiazine at our institution are the face, ears, and genitalia. As the most likely allergic reaction would be erythema and edema based on prior case reports [13, 14], it is possible that subtle signs of allergic reaction could have been missed, as they may be difficult to distinguish from the natural burn course. This is especially true given the retrospective nature of the study, as staff would not have been on alert at the time.

## Conclusions

Silver sulfadiazine is commonly used for burn care due to its advantageous properties for wound healing and antimicrobial effects. There is little evidence and a paucity of literature regarding the safety of silver sulfadiazine use for burn patients with documented sulfa allergy. Sources expressing caution about silver sulfadiazine use in sulfa-allergic patients rely almost exclusively on case reports. We present evidence in our retrospective review that administration of topical silver sulfadiazine in patients with sulfa allergies can be done in a safe manner, without adverse allergic reactions. If silver sulfadiazine is used, providers should follow the patient during their inpatient stay or in an outpatient clinic to monitor for likely signs of allergic reaction, including erythema and edema at the site of exposure.
